# Causal effects of gut microbiota on erectile dysfunction: a two-sample Mendelian randomization study

**DOI:** 10.3389/fmicb.2023.1257114

**Published:** 2023-10-19

**Authors:** Yuyang Zhang, Yuxi Chen, Yangyang Mei, Renfang Xu, Hong Zhang, Xingliang Feng

**Affiliations:** ^1^Department of Urology, The First Affiliated Hospital of Anhui Medical University, Hefei, Anhui, China; ^2^Department of Statistics and Finance, School of Management, University of Science and Technology of China, Hefei, Anhui, China; ^3^Department of Urology, Jiangyin People's Hospital of Jiangsu Province, Jiangyin, China; ^4^Department of Urology, The Third Affiliated Hospital of Soochow University, Changzhou, Jiangsu, China; ^5^Department of Urology, First People's Hospital of Changzhou, Changzhou, Jiangsu, China

**Keywords:** Mendelian randomization, gut microbiota, erectile dysfunction, causality, gut-penis axis

## Abstract

**Background:**

Several observational studies have reported the correlation between gut microbiota and the risk of erectile dysfunction (ED). However, the causal association between them remained unestablished owing to intrinsic limitations, confounding factors, and reverse causality. Therefore, the two-sample Mendelian randomization (MR) study was performed to determine the causal effect of gut microbiota on the risk of ED.

**Methods:**

The MR analysis utilized the publicly available genome-wide association study (GWAS) summary-level data to explore the causal associations between gut microbiota and ED. The gut microbiota data were extracted from the MiBioGen study (*N* = 18,340), and the ED data were extracted from the IEU Open GWAS (6,175 ED cases and 217,630 controls). The single nucleotide polymorphisms (SNPs) served as instrumental variables (IVs) by two thresholds of *P*-values, the first *P*-value setting as <1e-05 (locus-wide significance level) and the second *P*-value setting as <5e-08 (genome-wide significance level). The inverse variance weighted approach was used as the primary approach for MR analysis, supplemented with the other methods. In addition, sensitivity analyses were performed to evaluate the robustness of the MR results, including Cochran's Q test for heterogeneity, the MR-Egger intercept test for horizontal pleiotropy, the Mendelian randomization pleiotropy residual sum, and outlier (MR-PRESSO) global test for outliers, and the forest test and leave-one-out test for strong influence SNPs.

**Results:**

Our results presented that the increased abundance of *Lachnospiraceae* at family level (OR: 1.265, 95% CI: 1.054–1.519), *Senegalimassilia* (OR: 1.320, 95% CI: 1.064–1.638), *Lachnospiraceae NC2004* group (OR: 1.197, 95% CI: 1.018–1.407), *Tyzzerella3* (OR: 1.138, 95% CI: 1.017–1.273), and *Oscillibacter* (OR: 1.201, 95% CI: 1.035–1.393) at genus level may be risk factors for ED, while the increased abundance of *Ruminococcaceae UCG013* (OR: 0.770, 95% CI: 0.615–0.965) at genus level may have a protective effect on ED. No heterogeneity or pleiotropy was found based on the previously described set of sensitivity analyses.

**Conclusion:**

Our MR analysis demonstrated that the gut microbiota had inducing and protective effects on the risk of ED. The results provide clinicians with novel insights into the treatment and prevention of ED in the future. Furthermore, our study also displays novel insights into the pathogenesis of microbiota-mediated ED.

## 1. Introduction

Erectile dysfunction (ED), also known as impotence, is defined as the inability to achieve and maintain a sufficient penile erection to complete satisfactory sexual intercourse during sexual activity and is currently a common male sexual disorder (Hatzimouratidis et al., 2010). A recent systematic review of ED prevalence in multiple countries reported a range of ED prevalence from 37.2 to 48.6% across countries (Goldstein et al., 2020). This study (Goldstein et al., 2020) also showed that the prevalence of self-reported ED in men aged 40–70 years (45.2%) had increased significantly (10.0–30.0%) compared to previous studies (Nicolosi et al., 2004; Ahn et al., 2007). ED is a multifactorial disease, and several high-quality studies have demonstrated that hypertension, hyperlipidemia, diabetes, metabolic syndrome, and some psychological disorders (depression, anxiety) increase the risk of ED (Thompson et al., 2005; Saigal et al., 2006; Clark et al., 2007; Quek et al., 2008; Inman et al., 2009; Ponholzer et al., 2010). When it concerns ED, the topic that cannot be skipped is cardiovascular disease (CVD). Over the past decade, many studies have shown that ED can be regarded as an early sign of CVD (Clark et al., 2007; Inman et al., 2009). A well-documented meta-analysis demonstrated that ED significantly increases the risk of CVD (Chung et al., 2011). Therefore, risk factors for CVD such as hypertension, dyslipidemia, diabetes, smoking, and obesity are also applicable to ED (Feldman et al., 1994; Dong et al., 2011).

In recent years, the gut microbiota has come to the forefront with the development and application of sequencing technologies and may play a key role in all aspects of human health (Zoetendal et al., 2008). Through the immune system, intestinal barrier function, and disease vulnerability pathways, gut microbes regulate the physiological balance of this host to achieve a healthy state (Kamada et al., 2013). In the course of research, various diseases such as atherosclerosis (Meng et al., 2021), diabetes (Wu et al., 2020), obesity (Le Chatelier et al., 2013), depression, and anxiety (Simpson et al., 2021) are closely associated with the gut microbiota. All of these diseases can induce the development of ED, so it is likely that ED is closely linked to gut microbiota. A recent review proposed a five-level chain of proof for microbial-associated diseases, from the beginning of correlation to the final molecular mechanistic study, called the “funnel” model (Chaudhari et al., 2021). However, there are few studies on the relationship between ED and gut microbiota. A cross-sectional study from a Japanese community demonstrates that the relative abundance of *Alistipes* and *Clostoridium XVIII* in the gut microbiota is an independent risk factor for poor erectile function (Okamoto et al., 2020). As the study progressed, another study further explored the relationship between ED and gut microbiota, showing that ED patients had a significantly different gut microbial composition than controls, and found that *Actinobacillus* may act as a pathogenic bacterium that is significantly negatively associated with erectile function (Kang et al., 2023).

Current cross-sectional studies only showed a strong correlation between ED and gut microbes, which remains at the most superficial level of evidence due to the influence of confounding factors and reverse causality that cannot account for a causal relationship between the two. To overcome this limitation, Mendelian randomization (MR), as an epidemiological approach, uses the random assignment of single nucleotide polymorphisms (SNPs) during conception to infer causality between exposure and outcome through genetic variation. The nature of this random assignment excludes confounding factors and gives MR the value of a randomized controlled trial (Davey Smith and Hemani, 2014; Davies et al., 2018; Richmond and Davey Smith, 2022). To date, no study has assessed the relationship between gut microbiota and ED by MR methods, and this study uses data from a large genome-wide association study (GWAS) to assess the causal relationship between ED and gut microbiota within the framework of MR for the first time.

## 2. Materials and methods

### 2.1. Study design and data resources

**Figure 1:**
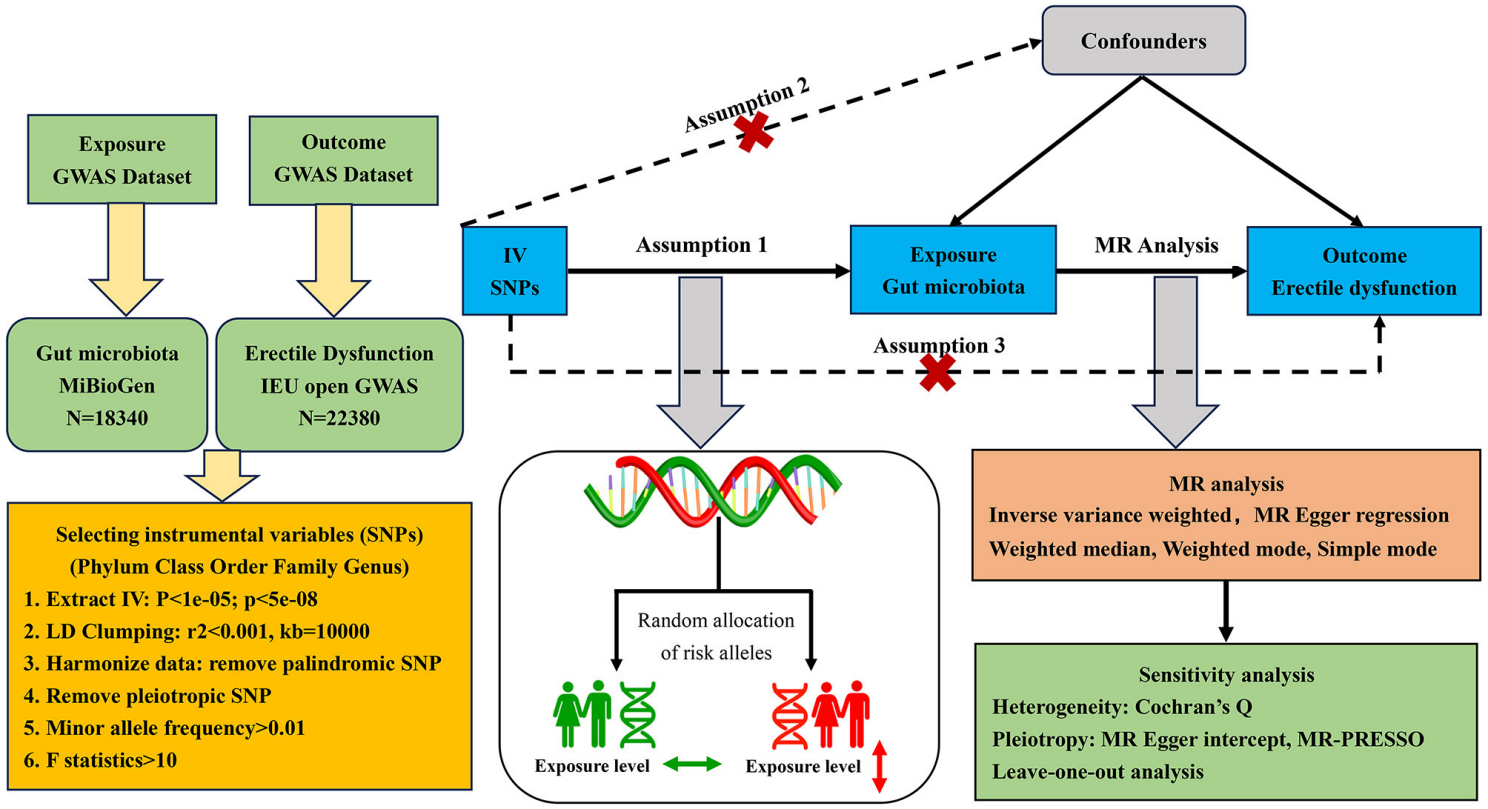
Flowchart of the MR randomization study and major assumptions. MR, Mendelian randomization; GWAS, genome-wide association study; SNPs, single nucleotide polymorphisms; IVW, inverse-variance weighted; LD, linkage disequilibrium; MR-PRESSO, MR pleiotropy residual sum and outlier.

The two-sample MR randomization study was conducted to explore the causal role of gut microbiota on the risk of ED in accordance with the STROBE-MR guidelines (Skrivankova et al., 2021), and the checklist is available in Supplementary Table S1. The detailed flowchart of our study is presented in Figure 1. In brief, the genetic variants strongly associated with the exposure factors were extracted from GWAS summary-level data and used as IVs (Burgess et al., 2017). A total of five MR methods were conducted for the two-sample MR analysis sequentially, as the inverse variance weighted (IVW) test served as the primary analysis approach supplemented by the other methods, including MR-Egger regression, weighted median, weighted mode, and simple mode. Then, a set of sensitivity analyses were performed for essential associations, including the heterogeneity test, pleiotropy test, and leave-one-out analysis. Additionally, there were three core assumptions of MR analysis that we sought to meet to decrease the effect of bias and improve the reliability of our findings (Slob and Burgess, 2020). First, all IVs should be significantly associated with the exposure factor, named relevance assumption; second, all IVs should be independent of other confounding factors that may affect exposure and outcome potentially, named independence assumption; and third, all IVs influence the outcome only through the exposure, named exclusion restriction assumption.

The gut microbiota GWAS summary-level data of interest were obtained from the MiBioGen study, the biggest and most multiracial genome-wide meta-analysis of gut microbiota composition. The MiBioGen study contained genome-wide genotyping data and 16SRNA gene sequencing profiles of a total of 18,340 individuals from 24 cohorts in the United States, the United Kingdom, Finland, Sweden, Denmark, the Netherlands, and other countries. Of them, most were of European descent (13,266 individuals). A total of 211 microbiota taxa were finally classified in the summary data of this study, including 9 phyla, 16 classes, 20 orders, 32 families, and 119 genera. However, there were 3 unknown families, and 12 unknown genera were excluded from our final MR analysis. All the detailed characteristics of microbiota data were presented in the original study (Kurilshikov et al., 2021).

**Figure 2:**
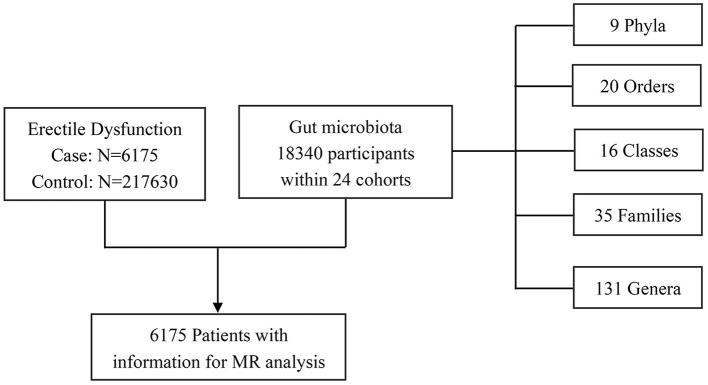
Study participants for the MR analysis. MR, Mendelian randomization.

The genetic data for ED were obtained from a recently published GWAS meta-analysis study of European ancestry. In brief, the study incorporated three cohorts from the Partners HealthCare Biobank, the Estonian Genome Center of the University of Tartu, and the UK Biobank. A total of 223,805 participants were enrolled in the combined cohort, including 6,175 ED cases and 217,630 controls, respectively. The ED diagnoses were confirmed based on the International Classification of Diseases version 10 (ICD-10) codes (N48.4 and F52.2), a medical intervention history for ED-like surgery (OPCS-4 codes: L97.1 and N32.6), or oral drugs (vardenafil/Levitra, tadalafil/Cialis, or sildenafil/Viagra), or self-report from the participants. The detailed information could be further accessed through the original articles (Bovijn et al., 2019). The selection process of study participants is exhibited in Figure 2. The respective institutional review boards have approved all the published GWAS. Only the GWAS summary-level data were extracted and used in our secondary MR analysis, so there was no need to obtain additional ethical approval.

### 2.2. Selection of instrumental variables

The microbiota taxa were categorized and analyzed at five taxonomic levels (phylum, class, order, family, and genus). To ensure robustness and accuracy of the causality between gut microbiota and ED risk, the SNPs were quality checked to obtain complaint IVs following the quality control procedures. (1). The SNPs were chosen as IVs when they met the relevance assumption of the MR study. Two thresholds of the relevant *P*-value were used to select SNPs (*P* < 1e-05 and *P* < 5e-08) (Sanna et al., 2019). (2) The clumping parameters were set to r^2^ < 0.001, and kb = 10,000 kb in order to ensure independence among the selected SNPs. Additionally, the clumping procedures were also intended to minimize the effect of linkage disequilibrium, which could violate random allele assignment and destroy the MR basis. (3) The palindromic and incompatible alleles would be disqualified from the final MR analysis through the harmonizing procedure. (4) The SNPs would also be deleted if they were unsatisfied with the independence assumption of the MR study, as they were significantly associated (*P* < 5e-08) with such confounding factors as diabetes, obesity, and cigarette consumption. All the selected SNPs were manually searched in the PhenoScanner GWAS database (Kamat et al., 2019). No eligible SNPs were found to be associated with the confounding factors. (5) The effect allele frequency of the selected SNPs should be above 0.01. (6) The F-statistics of selected SNPs should be above 10 to avoid weak instrument bias (Pierce et al., 2011). The F-statistics were calculated using the formula *R*^2^^*^(n-k-1)/k(1-R^2^*)* (Pierce et al., 2011). The n, k, and R^2^ of the formula represent the sample size, the number of SNPs, and the variance interpreted by the IVs, respectively.

### 2.3. Mendelian randomization analyses

Owing to the different thresholds of the *P*-values for selecting related SNPs, two sets of IVs were eligible for the MR analysis. When the gut microbiota feature contained only one SNP, the Wald ratio test was applied for analysis (Burgess et al., 2013); while the gut microbiota feature contained multiple SNPs, the aforementioned five methods would all be used for analysis. The IVW meta-analysis approach turns the outcome effects of the IVs on the exposure effects into a weighted regression with the intercept set to zero. The IVW technique assumed that there was no horizontal pleiotropy and could provide unbiased estimates by avoiding the effects of confounders (Holmes et al., 2017). Furthermore, we also conducted the Benjamini and Hochberg false discovery rate (FDR) to correct our results for multiple hypothesis testing (Korthauer et al., 2019). The FDR-corrected *P*-value was also set as *P* < 0.05. Consequently, the *P*-values were lower than 0.05, while the FDR-corrected *P*-values were larger than 0.05, so the association should be suggestive. All the two *P*-values were lower than 0.05, so the causality should be confirmed. As for the MR-Egger regression approach, its estimates would be heavily affected by outlier genetic variables (Bowden et al., 2016a). When at least 50% of the data from valid instruments were available, the weighted median technique could provide precise and reliable MR estimates (Bowden et al., 2016b). The weighted mode method is adaptable if the genetic variable defies the pleiotropy hypothesis (Hartwig et al., 2017). The simple mode method could also provide robustness for pleiotropy, although it is less efficient than the IVW method (Milne et al., 2017). The outcome of interests was identified as a binary variable, and all the MR estimates were expressed as odds ratios (OR) and 95% confidential intervals (CI).

### 2.4. MR sensitivity analysis

The heterogeneity and pleiotropy of IVs for MR analysis could seriously bias the results. Consequently, it is necessary to perform sensitivity analyses to verify the robustness of our significant results. Cochran's Q test of IVW and the MR-Egger approach were used to detect the IV heterogeneity, with a *P*-value of >0.05 indicating the lack of heterogeneity. Although we have manually searched the enrolled SNPs in the PhenoScanner GWAS database and excluded the potentially pleiotropic SNPs, which were significantly related to other confounding factors affecting ED risk independent of gut microbiota. The MR Egger intercept and Mendelian randomization pleiotropy residual sum and outlier (MR-PRESSO) global test were also further conducted to evaluate the potential horizontal pleiotropic effects of enrolled IVs. Additionally, to confirm the accuracy and robustness of the causal effect estimates, a leave-one-out sensitivity analysis was performed to evaluate the presence of strong influence SNPs on MR estimates. Additionally, we also performed the MR Steiger directionality test to infer causal direction (Hemani et al., 2017). Only when the variance explained by the IVs on the exposure of interest is greater than the outcome should the qualified causal link between exposure and outcome be considered directionally credible. The power computations were administered at the site (Brion et al., 2013).

For the evidence of significant causal effects, all the *P*-values were set at <0.05. All the statistical analyses were carried out using the publicly available R computational environment (version 4.1.2). The R packages “TwoSampleMR” and “MRPRESSO” were used for MR analysis and sensitivity analysis, respectively.

## 3. Results

### 3.1. MR estimate of gut microbiota on ED

**Figure 3:**
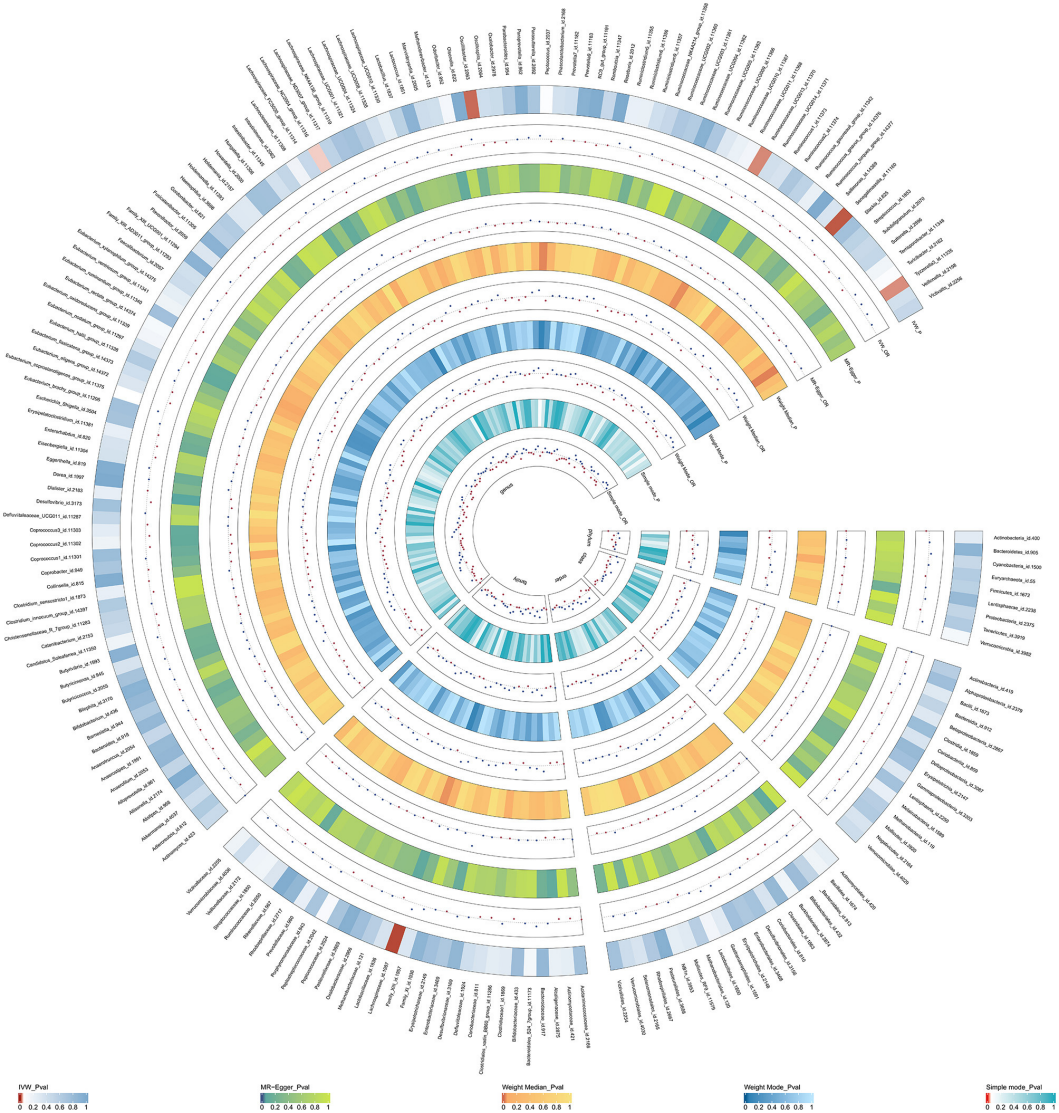
The circus plot showing the MR results of all gut microbiota. IVW, inverse-variance weighted; OR, odds ratio.

**Figure 4:**
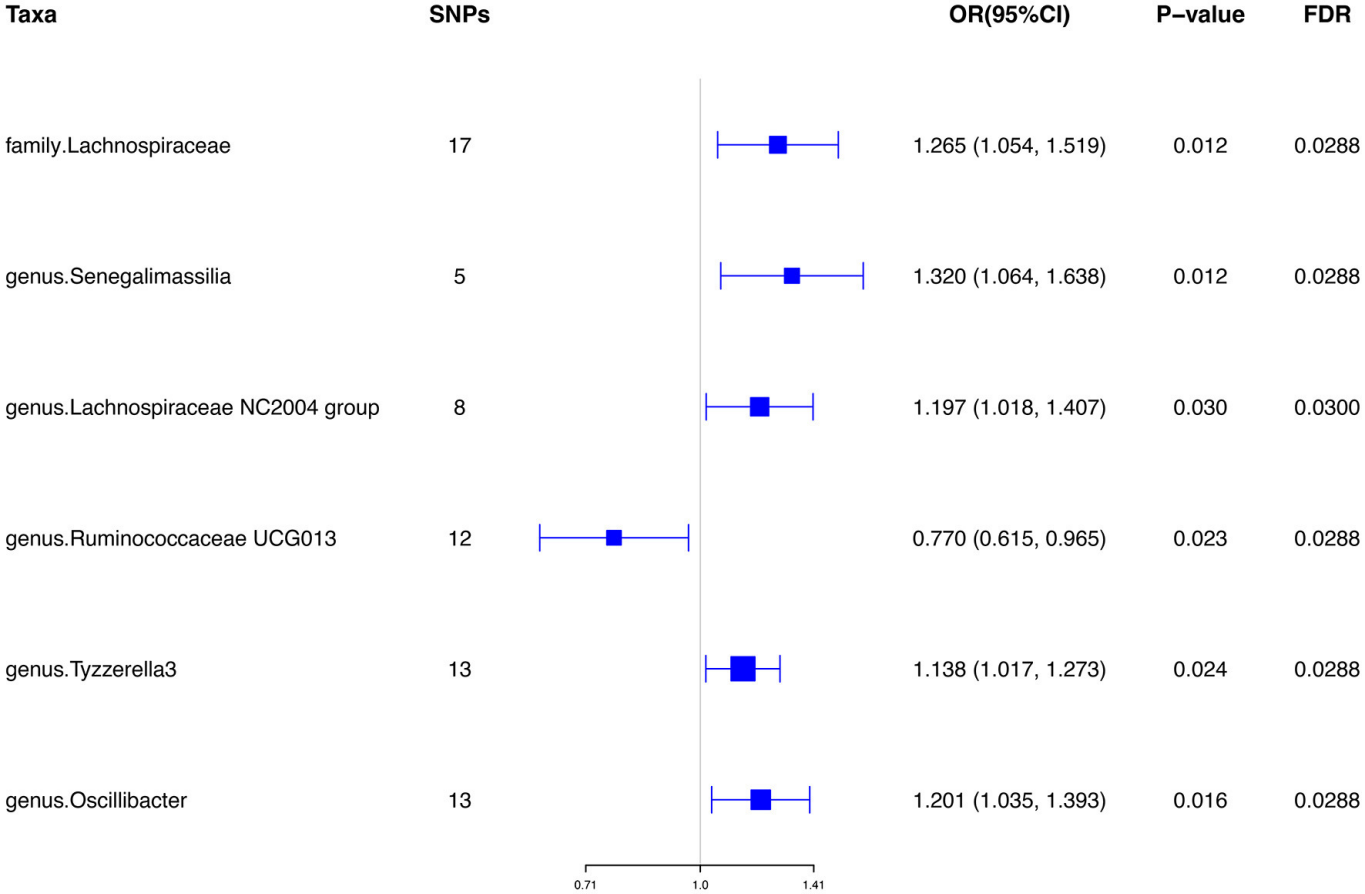
Significant results of the associations between genetically predicted gut microbiota and ED risk using IVW methods. IVW, inverse-variance weighted.

**Figure 5:**
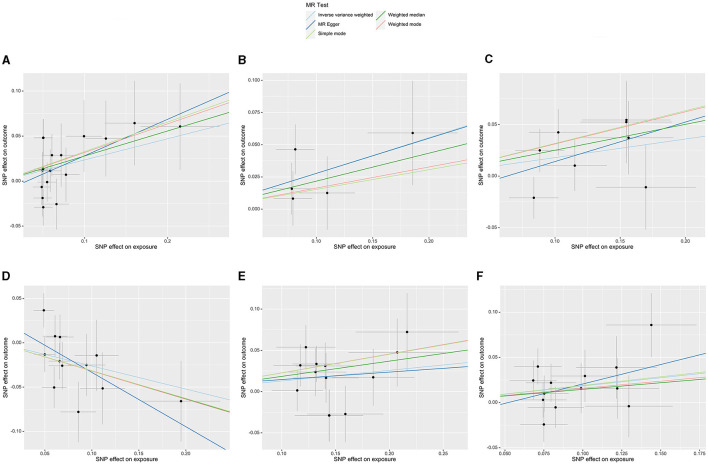
Scatterplots of the casual effect of gut microbiota on ED risk. **(A)** Family *Lachnospiraceae*, **(B)** genus *Senegalimassilia*, **(C)** genus *Lachnospiraceae NC2004* group, **(D)** genus *Ruminococcaceae UCG013*, **(E)** genus *Tyzzerella3*, and **(F)** genus *Oscillibacter*.

Initially, a total of 14,587 SNPs were identified as genetic instruments at the locus-wide significance level (*P* < 1e-05). These SNPs were correlated with the corresponding 211 gut microbiota traits, which include 9 phyla, 16 classes, 20 orders, 35 families, and 131 genera. The detailed characteristics of the eligible SNPs are presented in Supplementary Table S2, which includes the effect allele, other allele, beta, se, *P*-value, and corresponding sample size. The MR analyses were performed for each pair of exposure (microbiota taxa) and outcome (ED) using the qualified SNPs passing a series of filter criteria described above. All the MR causal associations between 196 gut microbiota and ED risk are shown in Figure 3, including the P-values and OR values. Specifically, the results of the IVW approach reaching the significant threshold of *P* < 0.05 are presented in Figure 4. The results of the IVW analysis elucidated that causal effects of the genetically predicted increased abundance of *Lachnospiraceae* (OR: 1.265, 95% CI: 1.054–1.519) at family level and *Senegalimassilia* (OR: 1.320, 95% CI: 1.064–1.638), *Lachnospiraceae NC2004* group (OR: 1.197, 95% CI: 1.018–1.407), *Tyzzerella3* (OR: 1.138, 95% CI: 1.017–1.273), and *Oscillibacter* (OR: 1.201, 95% CI: 1.035–1.393) at genus level were associated with the higher risk of ED, while the increased abundance of *Ruminococcaceae UCG013* (OR: 0.770, 95% CI: 0.615–0.965) at genus level were associated with the lower risk of ED. The results of the remaining four methods were consistent in direction with the IVW analysis, which reinforced the causal effect of gut microbiota on the risk of ED. All the detailed information about these statistically significant results is displayed in Table 1. In addition, the scatterplots are presented in Figure 5, reflecting the causal association between the corresponding gut microbiota and ED.

**Table 1:** MR estimates for the association between gut microbiota and ED (*p* < 1 × 10^−5^).

**Level**	**Microbiota**	**SNPs**	**R^2^ (%)**	**Methods**	**Beta**	**OR (95%CI)**	***P*-value**
Family	Lachnospiraceae	17	2.5	Inverse variance weighted	0.235	1.265 (1.054, 1.519)	0.012
				MR Egger	0.405	1.500 (0.954, 2.358)	0.099
				Weighted median	0.278	1.321 (1.030, 1.694)	0.028
				Weighted mode	0.318	1.374 (0.971, 1.944)	0.092
				Simple mode	0.327	1.387 (0.920, 2.091)	0.137
Genus	Senegalimassilia	5	1.6	Inverse variance weighted	0.277	1.320 (1.064, 1.638)	0.012
				MR Egger	0.273	1.313 (0.620, 2.783)	0.528
				Weighted median	0.217	1.243 (0.937, 1.647)	0.131
				Weighted mode	0.163	1.177 (0.822, 1.687)	0.423
				Simple mode	0.153	1.166 (0.805, 1.688)	0.463
	Lachnospiraceae NC2004 group	8	2.8	Inverse variance weighted	0.179	1.197 (1.018, 1.407)	0.030
				MR Egger	0.383	1.466 (0.766, 2.806)	0.292
				Weighted median	0.251	1.285 (1.031, 1.602)	0.026
				Weighted mode	0.312	1.366 (0.957, 1.948)	0.129
				Simple mode	0.317	1.373 (0.961, 1.961)	0.125
	Ruminococcaceae UCG013	12	1.7	Inverse variance weighted	−0.261	0.770 (0.615, 0.965)	0.023
				MR Egger	−0.612	0.542 (0.296, 0.994)	0.076
				Weighted median	−0.317	0.728 (0.550, 0.965)	0.027
				Weighted mode	−0.318	0.727 (0.506, 1.046)	0.114
				Simple mode	−0.313	0.731 (0.478, 1.119)	0.177
	Tyzzerella3	13	5.9	Inverse variance weighted	0.129	1.138 (1.017, 1.273)	0.024
				MR Egger	0.090	1.094 (0.581, 2.061)	0.786
				Weighted median	0.185	1.203 (1.033, 1.401)	0.017
				Weighted mode	0.228	1.256 (0.988, 1.596)	0.087
				Simple mode	0.226	1.253 (0.958, 1.639)	0.125
	Oscillibacter	13	3.4	Inverse variance weighted	0.183	1.201 (1.035, 1.393)	0.016
				MR Egger	0.429	1.536 (0.808, 2.920)	0.217
				Weighted median	0.146	1.157 (0.947, 1.414)	0.152
				Weighted mode	0.157	1.170 (0.801, 1.710)	0.432
				Simple mode	0.190	1.210 (0.850, 1.720)	0.311

MR, Mendelian randomization; ED, erectile dysfunction; SNPs, single nucleotide polymorphisms; R^2^, the proportion of phenotypic variation explained by the SNPs; OR, odds ratio; CI, confidence interval; MR–Egger, Mendelian randomization–Egger.

However, only 1,394 SNPs were qualified as genetic instruments at the genome-wide significance level (*P* < 5e-08). The SNPs were selected for MR analysis without linkage disequilibrium effects. The detailed characteristics of the selected SNPs are given in Supplementary Table S5. No causal effects were identified by the IVW analysis (OR: 1.028, 95% CI: 0.879, 1.202) when considering the gut microbiota as a whole. When the gut microbiota was treated as individual microbiota, the MR analysis showed the genetically predicted increased abundance of *Gastranaerophilales* (OR: 1.874, 95% CI: 1.291, 2.719) in order level was related to the higher risk of ED, while *Romboutsia* (OR: 0.388, 95% CI: 0.224, 0.673) in genus level was negatively related to the risk of ED. However, no other gut microbiota features identified have causal effects on the risk of ED. The results are shown in Table 2.

**Table 2:** MR estimates for the association between gut microbiota and ED (*p* < 5 × 10^−8^).

**Level**	**Microbiota**	**SNPs**	**Methods**	**Beta**	**SE**	**OR (95%CI)**	***P*-value**
Total		13	Inverse variance weighted	0.027	0.080	1.028 (0.879, 1.202)	0.732
			MR Egger	0.048	0.284	1.049 (0.601, 1.831)	0.869
			Weighted median	0.134	0.088	1.144 (0.963, 1.358)	0.125
			Weighted mode	0.174	0.140	1.190 (0.905, 1.565)	0.236
			Simple mode	0.156	0.120	1.168 (0.924, 1.477)	0.218
Phylum	Actinobacteria	1	Wald ratio	0.358	0.257	1.430 (0.864, 2.368)	0.164
Class	Actinobacteria	1	Wald ratio	0.160	0.209	1.173 (0.779, 1.767)	0.445
Order	Bifidobacteriales	1	Wald ratio	0.189	0.185	1.208 (0.840, 1.737)	0.309
	Gastranaerophilales	1	Wald ratio	0.628	0.190	1.874 (1.291, 2.719)	0.001
Family	Bifidobacteriaceae	2	Inverse variance weighted	0.189	0.185	1.208 (0.840, 1.737)	0.309
	Oxalobacteraceae	1	Wald ratio	0.096	0.182	1.101 (0.771, 1.572)	0.596
	Peptostreptococcaceae	1	Wald ratio	−0.957	0.284	0.384 (0.220, 0.670)	0.001
	Streptococcaceae	1	Wald ratio	−0.002	0.313	0.998 (0.541, 1.842)	0.996
Genus	Eubacteriumcoprostanoligenesgroup	1	Wald ratio	0.030	0.319	1.031 (0.551, 1.926)	0.925
	Ruminococcustorquesgroup	1	Wald ratio	0.181	0.322	1.199 (0.638, 2.253)	0.573
	Allisonell	1	Wald ratio	−0.171	0.130	0.843 (0.654, 1.087)	0.187
	Bifidobacterium	2	Inverse variance weighted	0.184	0.181	1.203 (0.843, 1.716)	0.309
	Erysipelatoclostridium	1	Wald ratio	0.143	0.231	1.154 (0.734, 1.814)	0.536
	Intestinibacter	1	Wald ratio	0.169	0.306	1.184 (0.650, 2.156)	0.580
	Oxalobacter	1	Wald ratio	0.244	0.170	1.277 (0.916, 1.780)	0.150
	Romboutsia	1	Wald ratio	−0.947	0.281	0.388 (0.224, 0.673)	0.001
	RuminococcaceaeUCG013	1	Wald ratio	−0.315	0.314	0.729 (0.394, 1.350)	0.315
	Streptococcus	1	Wald ratio	−0.001	0.297	0.999 (0.558, 1.786)	0.996
	Tyzzerella3	1	Wald ratio	0.253	0.166	1.288 (0.931, 1.783)	0.126

MR, Mendelian randomization; ED, erectile dysfunction; SNPs, single nucleotide polymorphisms; R^2^, the proportion of phenotypic variation explained by the SNPs; OR, odds ratio; CI, confidence interval; MR–Egger, Mendelian randomization–Egger.

### 3.2. Sensitivity analysis of MR estimates

The F-statistics of all selected SNPs at the locus-wide significance level were all larger than 10, indicating the absence of the weak IV bias (Supplementary Table S3). Cochran's Q test of the MR-Egger and IVW approaches showed no significant heterogeneity among these SNPs. Furthermore, all the *P*-values of the MR Egger intercept and MR-PRESSO global test were greater than 0.05, indicating the absence of directional horizontal pleiotropy. The MR-PRESSO analyses were unable to identify any potential outliers. The sensitivity analysis and horizontal pleiotropy analysis results are presented in Table 3. Of note, all the MR Steiger directionality results were more than 0.05, indicating that all six causal effects from gut microbiota to ED were robust in direction. The power results showed that the power to evaluate the causal effects of these microbiota features on ED was satisfied; most of them were >70% (Supplementary Table S4). No strong single SNP was identified to drive the MR estimation by the forest plots and leave-one-out test (Supplementary Figures S1, S2). All these sensitivity analyses reinforced the robustness of our findings.

**Table 3:** Evaluation of heterogeneity and directional pleiotropy using different methods.

		**Heterogeneity**		**Horizontal pleiotropy**	
**Level**	**Microbiota**	**Q_*****P*** **value (IVW)**	**Q_*****P*** **value (MR Egger)**	**MR egger intercept P**	**MR-PRESSO global test P**
Family	Lachnospiraceae	0.369	0.347	0.431	0.421
Genus	Senegalimassilia	0.653	0.484	0.990	0.694
	Lachnospiraceae NC2004 group	0.466	0.397	0.549	0.498
	Ruminococcaceae UCG013	0.239	0.278	0.251	0.280
	Tyzzerella3	0.506	0.423	0.905	0.539
	Oscillibacter	0.523	0.488	0.456	0.552

Q, Cochran's Q; IVW, inverse variance weighted; MR–Egger, Mendelian randomization–Egger; MR-PRESSO, Mendelian randomization pleiotropy residual sum and outlier.

The F-statistics of all selected SNPs at the genome-wide significance level were also larger than 10. When considering the gut microbiota as a whole, the results of Cochran's Q test revealed significant heterogeneity among these selected SNPs (MR-Egger: *P* = 0.03, IVW: *P* = 0.05). Of note, there was also no significant horizontal pleiotropy for the results of the MR-Egger intercept analysis (*P* = 0.94) and the MR-PRESSO analysis (*P* = 0.06). However, no further sensitivity analyses were undertaken as there were fewer SNPs for individual microbiota abundance.

## 4. Discussion

To the best of our knowledge, our study was the first two-sample MR analysis to meticulously evaluate the potential causal association between gut microbiota and ED through leveraging large-scale summary statistics of microbiota GWAS and ED GWAS. Our results showed a total of six microbiota features play an essential role in the development of ED. Among them, the increased abundance of *Lachnospiraceae, Senegalimassilia, Lachnospiraceae NC2004* group, *Tyzzerella3*, and *Oscillibacter* (OR>1, *P* < 0.05) may be risk factors for ED, while the increased abundance of *Ruminococcaceae UCG013* (OR < 1, *P* < 0.05) may have a protective effect on ED.

Trillions of symbiotic gut microbiomes are densely populated on the gastrointestinal mucosal surface of the host and serve as the natural barrier of the human body. The gut microbiota could regulate the balance between host health and sickness, and clinical diseases occur once the balance is disturbed. There is growing evidence that disturbance of the gut microbiome predisposes the host to multiple disorders, such as obesity (Le Chatelier et al., 2013), diabetes (Wu et al., 2020), atherosclerosis (Meng et al., 2021), and psychological disorders (Simpson et al., 2021). These conditions influenced by the gut microbiome were precisely associated with the occurrence of ED, which is mainly affected by vascular, hormonal, and psychological factors (Shamloul and Ghanem, 2013). Based on these theories, it is reasonable to correlate the disturbance of the gut microbiota with the risk of ED. A previous cross-sectional study in Japan failed to reveal a significant difference in the relative abundance of major bacterial genera between ED patients and individuals. However, significant differences between some species were found when they compared the composition of the gut microbiota between the low-ED group and the high-ED group, grouped based on IIEF-5 (Okamoto et al., 2020). Another study comparing the fecal bacterial diversity between ED patients and healthy controls also demonstrated that the ED group had lower bacterial diversity (Geng et al., 2021). Additionally, a pilot study not only compared the gut microbiome composition between ED patients and healthy men but also attempted to correlate the gut microbiome with the changes in erectile function, measured by the objective rigidity assessment system (Kang et al., 2023). However, another study conducted by Osman et al. (2023) failed to find a significant association between ED patients and age-matched health controls. As the authors declared, a small sample size may account for their results. Actually, no systematic analyses were conducted to specifically elucidate the roles of different gut microbiota in the incidence of ED. Our MR analysis yielded credible evidence at the genetic level.

Among the gut microbiota that have causal effects on the risk of ED, the family *Lachnospiraceae*, genus *Senegalimassilia*, genus *Lachnospiraceae NC2004* group, genus *Tyzzerella3*, and genus *Oscillibacter* increase the risk of ED. No previously published studies have reported the direct relationship between them and the risk of ED. However, several studies have demonstrated that they all play an important role in metabolic disorders like lipid, glucose, and obesity. Ley et al. found that the increased abundance of *Lachnospiraceae* was associated with a high body mass index (*P* = 0.002) (Ley et al., 2006). Another interesting study found that participants with a high intake of saturated fatty acids, a diet related to obesity (Chiu et al., 2017), showed an increased abundance of *Lachnospiraceae* and increased weight gain (Bailén et al., 2020). Another population cohort study revealed the role of *Tyzzerella3* in the occurrence of incident type 2 diabetes (Ruuskanen et al., 2022). A study also found that obese individuals with type 2 diabetes presented an increasing abundance of the genus *Oscillibacter* (Thingholm et al., 2019). There was less evidence reporting the correlation between the genus *Senegalimassilia* and the genus *Lachnospiraceae NC2004* group and metabolic disorders, therefore more research is needed. Although ample studies have explored their role in the progression of metabolic disorders, no specific mechanisms were found to explain the correlations. Therefore, in-depth studies of the mechanisms of the host-genetic drive associated with ED are critically required.

Except for their role in the metabolic disorders resulting in ED sequentially, some risky microbiota could also induce ED via their systematic pro-inflammatory roles. A previous study explored that enriched *Tyzzerella* in the gut was positively related to high CVD risk (Kelly et al., 2016). Another study by Zhang and their colleagues found that the family *Lachnospiraceae* was enriched in patients with type 2 diabetes when compared to normal controls (Zhang et al., 2013). The basic studies were consistent with these clinical studies. An animal study reported that polysaccharides could exert anti-inflammatory effects on high-fat diet-induced obese mice by depletion of *Oscillibacter* (Zhu et al., 2022). Another basic study also found that the low-cellulose diet could prompt gut inflammation by increasing the abundance of *Oscillibacter* (Zhang et al., 2013). Our results also verified their risky role in the occurrence of ED.

Several basic studies revealed that the genus *Ruminococcaceae UCG013* plays a protective role in hypertension. They reported that cold exposure could result in hypertension by decreasing the abundance of the genus *Ruminococcaceae UCG013* (Wang et al., 2022). The genus *Ruminococcaceae UCG013* belongs to the *Ruminococcaceae* family, known as butyrate-producing bacteria. Butyrate could exert anti-pro-inflammatory effects, and decreased butyrate-producing bacteria could induce inflammatory diseases such as hypertension, diabetes, and inflammatory bowel diseases (Bach Knudsen et al., 2018). Another study also found that the genus *Ruminococcaceae UCG013* was identified as the most significant biomarker for alleviating obesity (Feng et al., 2022). All these studies verified the protective role of *Ruminococcaceae UCG013* in inflammatory and metabolic diseases. Therefore, there is an urgent need to explore the role of gut microbiota in the prevention and treatment of ED.

In summary, our results showed that the gut microbiota has inducing and protective effects on the risk of ED. Moreover, our sensitivity analysis showed that no heterogeneity was detected by our series analysis. Therefore, our Mendelian randomization analysis results were robust and reliable. However, it is important to explore the specific mechanisms underlying these effects. Further clinical and basic studies should be performed to elucidate the complex interplay between gut microbiota and ED.

There were multiple strengths in our study. First, we are the first study to explore the causal effects of gut microbiota on the risk of ED by MR analysis to date. Second, the MR analysis could lessen the bias caused by the inevitable confounders and potential reverse causality compared to conventional observational studies. Consequently, our analysis could provide more convincing evidence to support the causality of gut microbiota and ED. Third, the data used for gut microbiota and ED were obtained from the largest GWAS meta-analysis up to data, which could guarantee the strengths of IVs and improve the MR analysis power. In addition, multiple sensitivity analyses ensure the robustness of our findings. Surely, several limitations inevitably existed in our study. First, a small number of participants in the gut microbiota GWAS were of non-European descent, which may partially bias our results. Second, the relatively lenient threshold (*P* < 1e-05) was adopted to select IVs, since an extremely small number of IVs meet the strict threshold (5e-08). However, the MR analysis was conducted on the two sets of IVs. Third, our analysis was restricted to the genus level rather than the species level, owing to the minimal taxonomic level in the MiBioGen study. Finally, the summary-level data for ED lack detailed group information for ED pathogenesis, such as organic ED or psychologic ED; so, we could not perform subgroup analyses between gut microbiota and ED subtypes.

## 5. Conclusion

Our MR analysis demonstrated a causal association between gut microbiota and ED, including *Lachnospiraceae, Senegalimassilia, Lachnospiraceae NC2004* group, *Tyzzerella3, Oscillibacter*, and *Ruminococcaceae UCG013*. Our results provide clinicians with novel insights into the treatment and prevention of ED in the future. Physicians and researchers need to pay more attention to monitoring gut microbiota in ED patients and find more salutary taxa for male sexual function. However, more in-depth analyses also need to be conducted in the future based on more advanced large-scale studies with metagenomics sequencing. Additionally, basic studies are also needed in the future to explore the mechanisms of gut microbiota in male ED.

## Data availability statement

The original contributions presented in the study are included in the article/Supplementary material, further inquiries can be directed to the corresponding authors.

## Ethics statement

Ethical review and approval were not required for the study on human participants in accordance with the local legislation and institutional requirements. Written informed consent for participation was not required for this study in accordance with the national legislation and the institutional requirements. The studies were conducted in accordance with the local legislation and institutional requirements. The participants provided their written informed consent to participate in this study.

## Author contributions

YZ: Conceptualization, Data curation, Formal analysis, Investigation, Writing—original draft. YC: Formal analysis, Investigation, Methodology, Software, Writing—original draft. YM: Conceptualization, Formal analysis, Investigation, Writing—review and editing. RX: Conceptualization, Investigation, Writing—review and editing. HZ: Formal analysis, Project administration, Software, Supervision, Writing—review and editing. XF: Conceptualization, Data curation, Funding acquisition, Writing—original draft, Writing—review and editing.
