# Supplementary material for: Causal effects of gut microbiota on erectile dysfunction: a two-sample Mendelian randomization study
Source: Front Microbiol. 2023 Oct 19;14:1257114. doi: 10.3389/fmicb.2023.1257114 (PMC10620728; doi:10.3389/fmicb.2023.1257114)

**Supplementary Figure S1.** Forest plots of the causal effects of gut microbiota on the risk of ED.

(A)family *Lachnospiraceae*; (B)genus *Senegalimassilia*; (C)genus *Lachnospiraceae NC2004 group*; (D)genus *Ruminococcaceae UCG013*; (E)genus *Tyzzerella3*; (F)genus *Oscillibacter.*


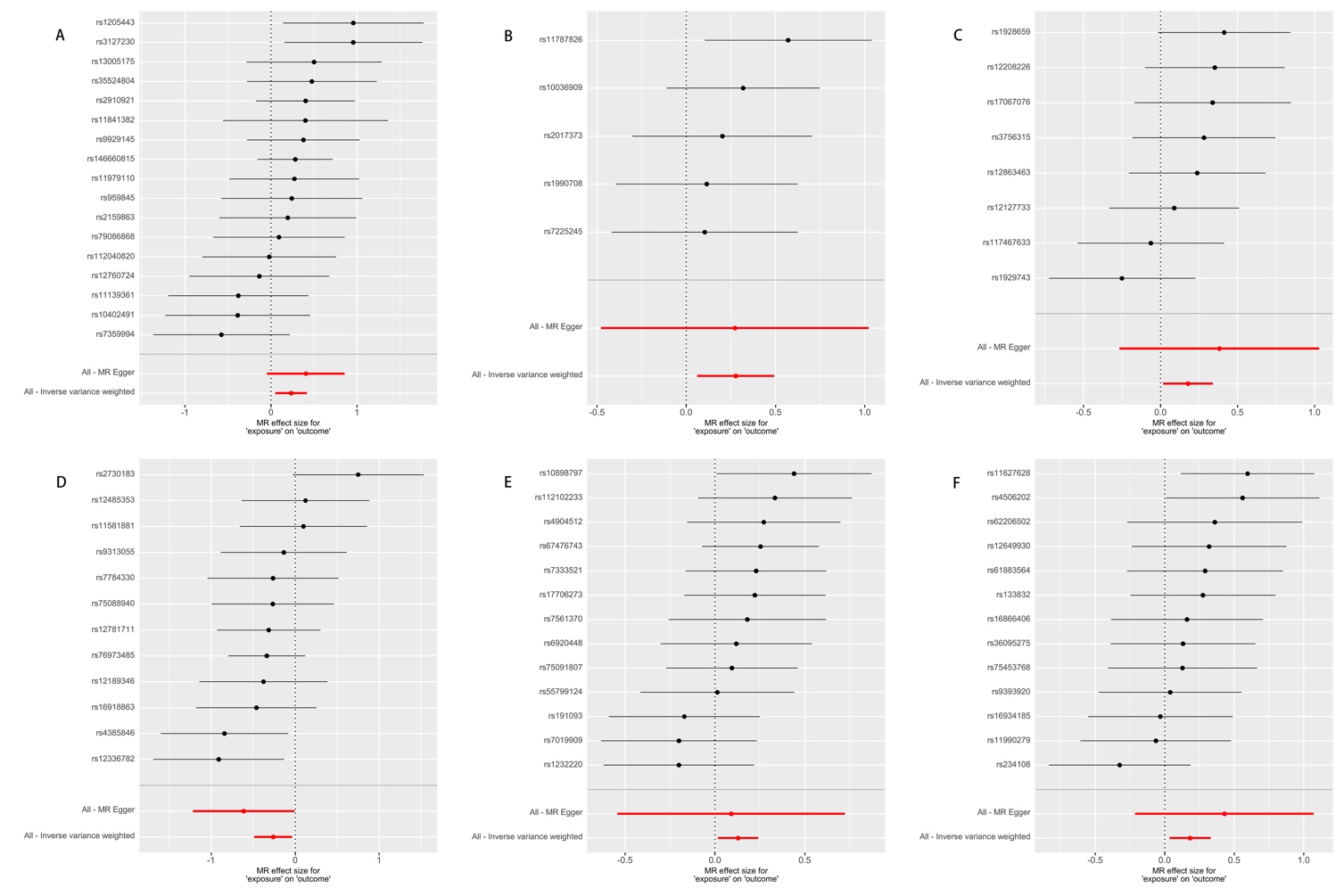


**Supplementary Figure S2.** Leave-one-out sensitivity analyses of the causal effects of gut microbiota on the risk of ED.

(A)family *Lachnospiraceae*; (B)genus *Senegalimassilia*; (C)genus *Lachnospiraceae NC2004 group*; (D)genus *Ruminococcaceae UCG013*; (E)genus *Tyzzerella3*; (F)genus *Oscillibacter.*


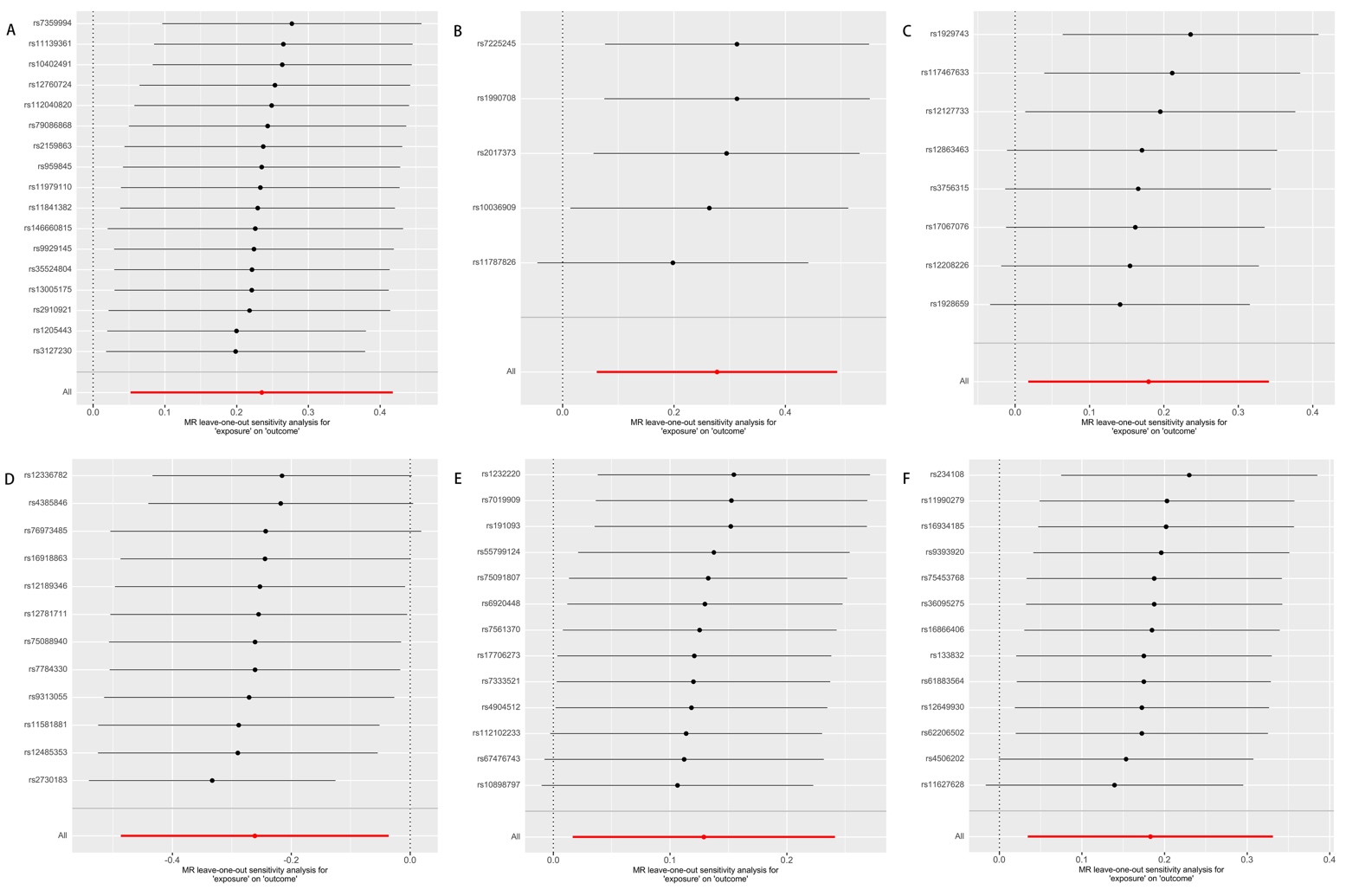

Supplement: Supplementary file 2 [file Data_Sheet_1.docx]
